# Eosinophilic esophagitis and risk of incident major adverse cardiovascular events: a nationwide matched cohort study

**DOI:** 10.1007/s10388-024-01066-8

**Published:** 2024-05-29

**Authors:** Anders Forss, Amiko M. Uchida, Bjorn Roelstraete, Fahim Ebrahimi, John J. Garber, Johan Sundström, Jonas F. Ludvigsson

**Affiliations:** 1 Department of Medical Epidemiology and Biostatistics, Karolinska Institutet, Box 281, 171 77 Stockholm, Sweden; 2 Centre for Digestive Health, Department of Gastroenterology, Dermatovenereology and Rheumatology, Karolinska University Hospital, Stockholm, Sweden; 3 Division of Gastroenterology, Hepatology and Nutrition, University of Utah School of Medicine, Salt Lake City, UT USA; 4 Department of Medicine, Division of Microbiology and Immunology, Department of Pathology, University of Utah School of Medicine, Salt Lake City, UT USA; 5 Department of Gastroenterology and Hepatology, Clarunis University Center for Gastrointestinal and Liver Diseases, Basel, Switzerland; 6 Gastrointestinal Unit, Massachusetts General Hospital, Harvard Medical School, Boston, MA USA; 7 Department of Medical Sciences, Uppsala University, Uppsala, Sweden; 8 The George Institute for Global Health, University of New South Wales, Sydney, NSW Australia; 9 Department of Paediatrics, Örebro University Hospital, Örebro, Sweden; 10 Department of Medicine, Columbia University College of Physicians and Surgeons, New York, NY USA

**Keywords:** Biopsy, Eosinophilic esophagitis, Epidemiology, Register-based

## Abstract

**Background:**

Inflammatory diseases have been associated with an increased cardiovascular risk. However, data on incident major adverse cardiovascular events (MACE) from large population-based cohorts of patients with eosinophilic esophagitis (EoE) is lacking.

**Methods:**

This study included all Swedish adults with EoE without a record of previous cardiovascular disease (CVD) (1990–2017, *N* = 1546) with follow-up until 2019. Individuals with EoE were identified from prospectively recorded histopathology reports from all Swedish pathology departments (*n* = 28). EoE patients were matched at index date for age, sex, calendar year and county with up to five general population reference individuals (*N* = 7281) without EoE or CVD. Multivariable-adjusted hazard ratios (aHRs) for MACE (ischemic heart disease, congestive heart failure, stroke and cardiovascular mortality) were calculated using Cox proportional hazards models. Full sibling comparisons and adjustment for cardiovascular medication were performed.

**Results:**

During a median follow-up of 6.0 years, we observed 65 incident MACE in patients with EoE (6.4/1000 person-years (PY)) and 225 in reference individuals (4.7/1000 PY). EoE was not associated with a higher risk of MACE (aHR = 1.14, 95% CI = 0.86–1.51) or any of its components. No differences between age, sex and follow-up time were observed. The results remained stable in sensitivity analyses, including when adjusting for relevant cardiovascular medications and a full sibling comparison.

**Conclusions:**

In this large population-based cohort study, patients with EoE had no increased risk of MACE compared to reference individuals and full siblings. The results are reassuring for patients with EoE.

**Supplementary Information:**

The online version contains supplementary material available at 10.1007/s10388-024-01066-8.

## Introduction

Eosinophilic esophagitis (EoE) is a chronic allergic inflammatory condition of the esophagus [1, 2]. Typical symptoms of EoE are dysphagia and recurrent food impactions, which cause impaired health-related quality of life and a substantial burden on the healthcare system [3, 4]. EoE affects both sexes but is more common among males. There is evidence of increasing EoE incidence and prevalence globally [5, 6]. Long-standing untreated EoE inflammation can lead to the development of fibrostenotic strictures or more diffuse narrowing of the esophagus [7]. Treatment of EoE includes diet elimination, proton pump inhibitors (PPI), topical corticosteroids and the newly approved anti-IL4Ra antibody (dupilumab) [8, 9].

There is evidence of an elevated risk of adverse cardiovascular events in patients with systemic inflammatory diseases [10]. Systemic inflammation has been suggested as a major driver of the atherosclerotic process, and anti-inflammatory compounds have been tested for the treatment of atherosclerotic cardiovascular disease (CVD) [11]. This association has been demonstrated also for other inflammatory gastrointestinal (GI) diseases [12, 13]. Although cardiovascular complications have long been recognised as a major cause of morbidity and mortality in hypereosinophilic syndrome [14], the risk of major adverse cardiovascular events (MACE) has not been investigated in patients with EoE. We hypothesized that eosinophilic inflammation in EoE, although limited to the esophagus, impacts the risk of CVD.

In this study, we aimed to investigate the risk of incident MACE in a nationwide histopathology cohort of all patients with biopsy-proven EoE in Sweden between 1990 and 2017 with follow-up until 2019.

## Methods

### Study population and case definitions

The Swedish healthcare system is primarily tax-funded. It offers universal access to health care to all residents. All Swedish healthcare providers (public and private) are mandated to report information to national administrative and healthcare registers.

This nationwide population-based matched cohort study used histopathology data in the ESPRESSO (Epidemiology Strengthened by Histopathology Reports in Sweden) cohort [15]. The ESPRESSO cohort includes prospectively recorded GI histopathology reports from all 28 pathology departments in Sweden (between the years 1965 and 2017). It has complete nationwide coverage of all GI histopathology (biopsy and surgical specimen) reports in Sweden. The reports contain data on biopsy date and location, description of topography within the GI tract and morphology coding according to the Systematised Nomenclature of Medicine (SNOMED) system. Biopsy data from the ESPRESSO cohort were linked through the unique personal identity number assigned to all Swedish legal residents to nationwide registers with data on demographics, migration, death, education level, medical diagnoses and prescribed medications. All patients in the ESPRESSO cohort were linked to and identified in those registers.

Outcome measures and covariates were ascertained from the Swedish National Patient Register (NPR) [16]. The NPR includes prospectively recorded inpatient data including discharge diagnoses and procedure codes since 1964, with full nationwide coverage from 1987, and since 2001 includes also outpatient visits (except primary care). Clinical diagnoses in the NPR show positive predictive values (PPVs) between 85 and 95%, including for CVD diagnoses [16].

We identified all individuals with a recorded esophageal biopsy in the ESPRESSO cohort submitted between 1965 and 2017 consistent with our definition of EoE diagnosis (Topography code: T62 (esophagus); SNOMED morphology code of inflammation that includes eosinophils: M47150) from patients of all ages at the date of biopsy (index date). We have previously validated this diagnostic algorithm for EoE and found a positive predictive value of 89% [17]. For this study, we excluded anyone with either a diagnosis of CVD or a record of emigration before EoE diagnosis (Table S1).

Each EoE patient was matched with up to five general population reference individuals identified from the Swedish Total Population Register (TPR) [18] with no diagnosis of EoE, CVD or earlier migration record at the time of the index biopsy date (hence identical exclusion criteria to those applied to EoE patients, Table S1). EoE patients and reference individuals were matched by age, sex, calendar year and county of residence at the index date. Patients with EoE and reference individuals were identified 1990–2017 and followed until 31 December 2019. The starting year 1990 was chosen as EoE awareness was low prior to this and diagnoses were few. A flowchart of inclusion and exclusion is presented in Figure S1.

### Outcomes and covariates

The primary outcome was incident MACE, defined as a composite outcome of ≥ 1 primary or secondary inpatient or hospital-based outpatient diagnosis of incident ischemic heart disease (IHD), congestive heart failure (CHF), stroke or CVD mortality as registered in the NPR after the index date (Table S2). Secondary outcomes were each of the individual MACE components. CVD mortality (as a primary cause of death) was ascertained from data in the Cause of Death Register [19]. In cases where one patient was diagnosed with more than one of the secondary outcomes, this patient would contribute to each outcome with the date of the relevant diagnosis.

Information was collected on demographics, educational level, comorbidities, and dispensed prescription medications for both EoE patients and reference individuals (Table S3). Age, sex, date of birth, residence at the index date, country of birth (two categories: Nordic, including Sweden, Denmark, Finland, Norway and Iceland, vs. Other countries) and emigration status were obtained from the TPR [18], while education level was ascertained from the longitudinal integrated database for health insurance and labour market studies [20]. Data on comorbidities were extracted from the NPR using ICD codes (Table S3) [16].

The Multigeneration Register (part of the TPR) was used to gather information on first-degree family members for sibling analyses.

We collected data on prescription medications for four different categories of medications (antiarrhythmics, vasodilators, anti-thrombotic medication and statins, Table S3) from the Prescribed Drug Register, a nationwide register with nearly full coverage [21] of data for dispensed prescriptions since 1 July 2005.

### Statistical analyses

Follow-up started at the index date (i.e., date of the first biopsy consistent with EoE for the exposed population and the corresponding matching date for the reference individuals). Follow-up continued to the first occurrence of any incident MACE outcome, non-CVD death, emigration or end of the follow-up (31 December 2019).

Incidence rates and absolute rate differences (RD) per 1000 person-years (PY) with 95% confidence intervals (95% CIs) were calculated for primary and secondary outcomes compared to reference individuals. We estimated multivariable-adjusted hazard ratios (aHRs) and 95% CIs for incident MACEs using Cox proportional hazards regression models. We did not account for competing events during follow-up. In a multivariable statistical model we stratified for matching factors, and adjusted for potential confounding risk factors for both EoE and MACE (including chronic respiratory disease, including asthma and chronic obstructive pulmonary disease (COPD) diagnosis, the latter as a proxy for heavy smoking) defined up to and including the index date [22]. We included both type 1 and 2 diabetes since it was not possible to distinguish between these types in ICD-8 or ICD-9. We also adjusted for a priori selected clinically relevant comorbidities (obesity, hypertension, dyslipidemia, chronic kidney disease, celiac disease, and atopic dermatitis) and educational level.

To account for potential confounding related to shared genetics and early-life environmental exposures, we performed sibling-controlled analyses. We compared EoE patients with ≥ 1 full sibling without a record of EoE or CVD before the start of the follow-up. We conditioned on the matching set within the family and adjusted for all covariates in the multivariable model.

We explored the effect of CVD medications on the associations between EoE and MACE in a restricted cohort of EoE patients and reference individuals between 1 January 2006 and 31 December 2019 (the start of follow-up was chosen to allow a washout period for prevalent medication and based on the availability of prescription medication data in the register) (Table S4). In addition to the covariates in the full cohort, we included dispensed prescriptions of four categories of CVD-related medications (Tables S3) at the start of the follow-up. These categories were formed to avoid an excessive number of covariates in the statistical modelling. In the restricted cohort.

The associations between EoE and MACE were investigated in stratified analyses according to sex, age, duration of follow-up, calendar period of the start of follow-up, country of birth (Nordic or Other countries), any metabolic disease (yes/no: composite variable consisting of diabetes, obesity, hypertension, and dyslipidemia). As a proxy for severe EoE, the restricted cohort was stratified by dispensed prescription of corticosteroids between 7 days before and up to 365 days after the index date. Corresponding analysis was performed also for PPIs.

The proportional hazards assumption was assessed through Schoenfeld residuals related to time. Two-sided *p*-values of < 0.05 were considered statistically significant. Statistical analyses were performed using R software (version 4.1.0, R Foundation for Statistical Computing, Vienna, Austria; survival package version 3.5 Therneau, 2015, https://CRAN.R-project.org/package=survival).

## Results

We included 1546 patients with biopsy-proven EoE and 7281 matched general population reference individuals. At the start of follow-up, the median age was 37 years (interquartile range (IQR) 19–51) for EoE patients. The majority of patients were males (75.0%). Atopic dermatitis and chronic respiratory disease (including COPD and asthma) were more frequent in EoE patients than in reference individuals. Additional baseline characteristics are presented in Table 1.

**Table 1:** Cohort characteristics for patients with eosinophilic esophagitis and general population reference individuals at start of follow-up 1990 with follow-up until 2019

Characteristics	Reference individuals	EoE
	*n* (%)	*n* (%)
Total	7 281	1 546
Sex		
Male	5 446 (74.8)	1 160 (75.0)
Female	1 835 (25.2)	386 (25.0)
Age (years)		
Mean (SD)	35.2 (19.0)	36.6 (19.7)
Median (IQR)	36 (18–50)	37 (19–51)
Range (Min–max)	0–90	0–90
Age-group (years)		
< 18	1 769 (24.3)	359 (23.2)
18 < 40	2 313 (31.8)	469 (30.3)
40 < 60	2 380 (32.7)	505 (32.7)
≥ 60	819 (11.2)	213 (13.8)
Country of birth		
Nordic	6 087 (83.6)	1 470 (95.1)
Other	1 193 (16.4)	76 (4.9)
NA	1 (0.0)	0 (0)
Educational level (years)		
Compulsory school (≤ 9)	1 073 (14.8)	233 (15.1)
Upper secondary school (10–12)	2 543 (34.9)	548 (35.4)
College or university (≥ 13)	2 288 (31.4)	487 (31.5)
NA	1 377 (18.9)	278 (18.0)
Start of follow-up period		
1990–1999	69 (0.95)	16 (1.03)
2000–2009	881 (12.10)	185 (11.97)
2010–2017	6 331 (86.95)	1 345 (87.00)
Follow-up (years)		
Mean (SD)	6.6 (3.0)	6.6 (3.0)
Median (IQR)	6.0 (4.6–8.0)	6.0 (4.6–8.0)
Range, min–max	0–28.3	0–26.6
0–1	157 (2.1)	27 (1.8)
2–9	6 259 (86.0)	1 342 (86.8)
≥ 10	865 (11.9)	177 (11.4)
Comorbidity at start of follow-up		
Any metabolic disease ≥ 1*	389 (5.3)	110 (7.1)
Diabetes	118 (1.6)	30 (1.9)
Obesity	108 (1.5)	19 (1.2)
Hypertension	207 (2.8)	68 (4.4)
Dyslipidemia	62 (0.9)	24 (1.6)
Kidney disease	102 (1.4)	28 (1.8)
Celiac disease	2 (0.03)	47 (3.0)
Atopic dermatitis	146 (2.0)	106 (6.9)
Inflammatory bowel disease	3 (0.04)	40 (2.6)
Chronic respiratory disease^†^	391 (5.4)	275 (17.8)

*EoE* Eosinophilic esophagitis; *IQR* interquartile range; *NA* data not available; *SD* standard deviation

*Includes ≥ 1 of diabetes, obesity, hypertension and dyslipidemia

^†^Includes chronic obstructive pulmonary disease and asthma

### Incident major adverse cardiovascular events

**Fig. 1:**
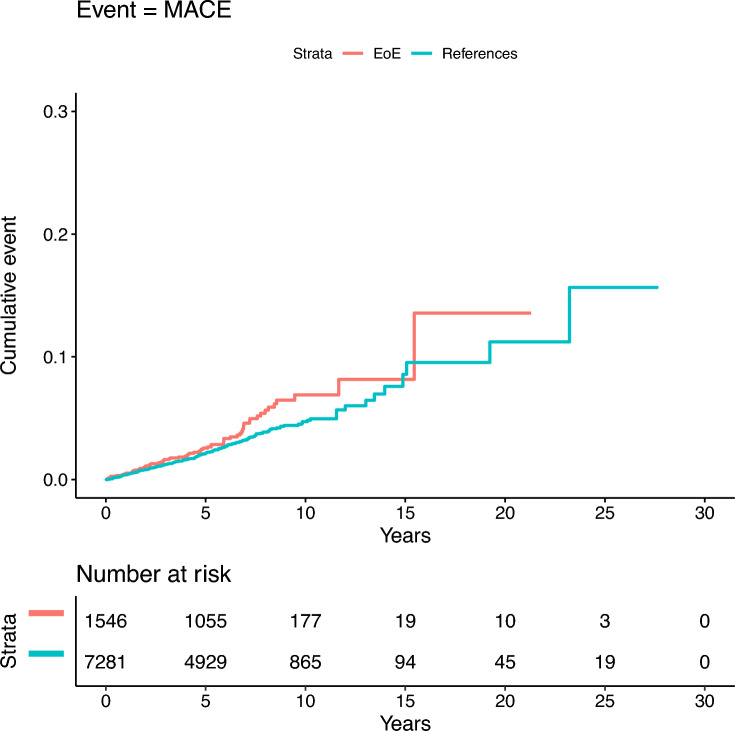
Cumulative incidence plot and 95% confidence intervals of overall incident major adverse cardiovascular events in patients with eosinophilic esophagitis and general population reference individuals 1990–2019

During a median follow-up of 6.0 years (IQR 4.6–8.0), we identified 65 incident MACE in patients with EoE (6.4/1000 PY, 95% CI = 4.9–8.1) and 225 in reference individuals (4.7/1000 PY, 95% CI = 4.1–5.4), corresponding to an absolute risk difference (RD) of 1.7/1000 PY (95% CI = 0.0–3.4) (Table 2, Fig. 1), and equivalent to one extra case of MACE for every 59 patients with EoE followed for 10 years. However, after multivariable adjustment, the association was not statistically significant (aHR: 1.14, 95% CI = 0.86–1.51) (Table 2). The cause-specific risks of the individual MACE components (IHD, CHF, stroke, and CVD mortality) were also not statistically significant (Table 3).

**Table 2:** Incidence rates, absolute rate differences and hazard ratios for incident major adverse cardiovascular events in patients with eosinophilic esophagitis compared to general population reference individuals 1990–2019

	Reference individuals	EoE
	*N* = 7 281	*N* = 1 546
MACE*		
Incident events (%)	225 (3.1)	65 (4.2)
Incidence rate per 1000 PY (95% CI)	4.7 (4.1–5.4)	6.4 (4.9–8.1)
Absolute rate difference per 1000 PY (95% CI)	0 (ref.)	1.7 (0.0–3.4)
Unadjusted HR (95% CI)	1 (ref.)	1.37 (1.04–1.80)
Adjusted HR^†^ (95% CI)	1 (ref.)	1.14 (0.86–1.51)

*CI* Confidence interval; *EoE* eosinophilic esophagitis; *HR* hazard ratio; *MACE* major adverse cardiovascular events; *PY* person-years

*Includes ischemic heart disease, congestive heart failure, stroke and cardiovascular mortality

^†^Adjusted for age, sex, calendar year, county of residence at index date, country of birth (Nordic country or other), educational level (compulsory school, upper secondary school or college/university), ≥ 1 metabolic disease (diabetes, obesity, hypertension or dyslipidemia), chronic kidney disease, celiac disease, atopic dermatitis and chronic respiratory disease (includes chronic obstructive pulmonary disease and asthma)

**Table 3:** Incidence rates, absolute rate differences and hazard ratios for incident ischemic heart disease, congestive heart failure, stroke and cardiovascular mortality in patients with eosinophilic esophagitis compared to general population reference individuals 1990–2019

Outcome	Reference individuals	EoE
	*N* = 7 281	*N* = 1 546
Ischemic heart disease		
Incident events (%)	108 (1.5)	34 (2.2)
Incidence rate per 1000 PY (95% CI)	2.2 (1.8–2.7)	3.3 (2.3–4.6)
Absolute rate difference per 1000 PY (95% CI)	0 (ref.)	1.1 (− 0.1–2.3)
Unadjusted HR (95% CI)	1 (ref.)	1.50 (1.02–2.20)
Adjusted HR* (95% CI)	1 (ref.)	1.28 (0.86–1.91)
Congestive heart failure		
Incident events (%)	57 (0.8)	24 (1.6)
Incidence rate per 1000 PY (95% CI)	1.2 (0.9–1.5)	2.3 (1.5–3.5)
Absolute rate difference per 1000 PY (95% CI)	0 (ref.)	1.1 (0.2–2.1)
Unadjusted HR (95% CI)	1 (ref.)	1.97 (1.22–3.17)
Adjusted HR* (95% CI)	1 (ref.)	1.62 (0.98–2.65)
Stroke		
Incident events (%)	67 (0.9)	13 (0.8)
Incidence rate per 1000 PY (95% CI)	1.4 (1.1–1.8)	1.3 (0.7–2.2)
Absolute rate difference per 1000 PY (95% CI)	0 (ref.)	− 0.1 (− 0.9–0.6)
Unadjusted HR (95% CI)	1 (ref.)	0.91 (0.50–1.64)
Adjusted HR* (95% CI)	1 (ref.)	0.78 (0.42–1.42)
Cardiovascular mortality		
Incident events (%)	41 (0.6)	8 (0.5)
Incidence rate per 1000 PY (95% CI)	0.8 (0.6–1.1)	0.8 (0.3–1.5)
Absolute rate difference per 1000 PY (95% CI)	0 (ref.)	− 0.1 (− 0.7 to 0.5)
Unadjusted HR (95% CI)	1 (ref.)	0.92 (0.43–1.96)
Adjusted HR* (95% CI)	1 (ref.)	0.70 (0.32–1.54)

*CI* Confidence interval; *EoE* eosinophilic esophagitis; *HR* hazard ratio; *MACE* major adverse cardiovascular events; PY, person-years

*Adjusted for age, sex, calendar year, county of residence at index date, country of birth (Nordic country or other), educational level (compulsory school, upper secondary school or college/university), ≥ 1 metabolic disease (diabetes, obesity, hypertension or dyslipidemia), chronic kidney disease, celiac disease, atopic dermatitis and chronic respiratory disease (includes chronic obstructive pulmonary disease and asthma)

In stratified analyses of MACE, no significant differences were found between the sexes, age groups, length of follow-up (< 5 vs. ≥ 5 years), periods of follow-up (starting in 1990), country of birth, or presence of the metabolic syndrome (Table S5).

### Sibling comparison

To account for potential confounding related to genetic risks and early-life environmental exposures, we compared 1178 EoE patients to their full-siblings (*n* = 1958) without a diagnosis of EoE or CVD at the index date. Similarly, in this analysis, EoE was not linked to any MACE (RD, 1.1/1000 PY; aHR = 1.33, 95% CI = 0.81–2.16) (Table S6).

### Sensitivity analyses

In a restricted cohort of EoE patients diagnosed since 2006 (*n* = 1508) we investigated the potential influence of CVD medication (four groups of medications) on MACE compared to reference individuals (*n* = 7106). Additional adjustment for CVD medication (at the start of follow-up), did not change the risk estimate (aHR = 1.07, 95% CI = 0.79–1.46) (Table S7). Stratifying the restricted cohort by ≥ 1 dispensed prescription of corticosteroids (inhalation/topical and oral, 7 days before and up to 365 days after the index date; yes/no), we saw no statistically significant differences between the groups (P-heterogeneity 0.52) (Table S8). Similarly, there were no differences between those exposed to PPIs and non-exposed (P-heterogeneity 0.98). We also compared those with neither corticosteroids or PPIs with those with corticosteroids and PPIs and found no significant difference (P-heterogeneity 0.50) (Table S8).

## Discussion

In this nationwide cohort of all Swedish individuals with biopsy-proven EoE (1990–2019) we found no increased risk of incident MACE compared to matched population reference individuals. No significant differences in sex, age group, country of birth or follow-up period were seen, and the results were consistent after adjustment for relevant CVD medications and in sibling analyses.

Epidemiological studies support the role of systemic inflammation in the development of CVD in different GI diseases [12, 13]. The large randomised clinical trial Canakinumab Anti-Inflammatory Thrombosis Study (CANTOS) [23] showed evidence that systemic inflammation increases atherosclerotic plaque formation, leading to adverse CVD events [24]. Hence, we hypothesised that the inflammatory burden in EoE might also share common pro-inflammatory features associated with an elevated risk of CVD outcomes. However, in the current study, we found no association between EoE and incident MACE after adjustment for CVD risk factors such as diabetes, dyslipidemia, obesity, hypertension, and chronic obstructive respiratory diseases. Our results were confirmed in full sibling comparisons, and in the sensitivity analysis where we adjusted for dispensed CVD medications.

We speculate that the systemic inflammatory burden in EoE is minimal, and therefore the link to atherosclerosis and CVD outcomes is weaker. When markers of general systemic inflammation such as C-reactive protein and serum cytokines have been investigated in EoE, to date most have not been associated with active systemic inflammation [25, 26], which is consistent with inflammation in EoE being relatively limited to the esophagus. This is in contrast with the systemic inflammation seen in hypereosinophilic syndrome where signs and symptoms of cardiac disease are present in approximately half of patients [27, 28]. Our findings also contrast with other chronic inflammatory GI diseases such as inflammatory bowel disease, where elevated inflammatory markers as signs of systemic inflammation, are common.

Since corticosteroid exposure is a known risk factor for CVD, and in patients with EoE could indicate a more severe disease, we performed stratified analysis to explore the potential effect of such exposure on our risk estimates. Interestingly, we found no statistically significant differences between patients with EoE exposed to corticosteroids and PPIs, or both, compared to non-exposed.

Furthermore, other atopic disorders have been linked to CVD, and asthma is a common comorbidity in EoE patients. In our study 17.8% of EoE patients and 5.4% of the reference individuals had chronic respiratory disease (defined as asthma or other obstructive pulmonary diseases such as COPD). A meta-analysis based on 18 studies, found a positive association between asthma and any CVD (relative risk (RR) = 1.33, 95% CI = 1.19–1.50), and CVD mortality (RR = 1.35, 95% CI = 1.15–1.59) [29]. The risk estimates were particularly high for heart failure (RR = 2.10, 95% CI = 1.98–2.22). Hence, we adjusted for asthma and did not see any increased risk of MACE outcomes in EoE patients compared to reference individuals.

To the best of our knowledge, we are not aware of any previous study that has investigated the associations between EoE and major CVD outcomes in a large population-based histopathology cohort. However, mortality in EoE patients was investigated in a Swedish population-based matched histopathology cohort study (*N* = 1625) [30]. Consistent with our findings, that study found no increased cause-specific risk of CVD-related death compared to general population reference individuals (aHR = 0.75, 95% CI = 0.32–1.79) [30].

Our study has several strengths, such as its nationwide study population, the high validity of EoE diagnosis, the components of MACE and several covariates. The study also benefited from nearly complete coverage of the involved healthcare registers. The comparably large cohort sample size allowed us to estimate not only the risks of MACE in several sensitivity analyses but also its components.

We acknowledge some limitations of our study. First, we cannot exclude that the reference population also included patients with undiagnosed EoE. However, EoE requires histopathological evidence for diagnosis and we, therefore, expect few false negatives and false positives. Second, the rather young study population and a median follow-up time of 6 years (IQR 5–8) may in some cases not have allowed the outcome to occur during the study period due to low CVD risk profiles. Third, we did not have data on important risk factors for EoE and MACE, such as detailed smoking data, body mass index, alcohol consumption, lipid profiles and dietary habits. Of note, our results continued to be robust in sensitivity analyses that included sibling analysis and adjustment for relevant CVD medications. Fourth, our data did not allow us to assess the severity of disease, and this included a lack of data on symptoms and symptom onset. Hence it was beyond the scope of this study to examine the role of diagnostic delay for the risk of MACE. In 2013, Schoepfer et al. reported a median delay of 6 years from symptom onset to diagnosis [7]. Future studies should examine the role of disease severity for CVD risk in EoE. Such measures may include patient symptoms, and quality of life, but also measures of inflammatory burden including cytokine levels, grading of mucosal lesions, as well as more granular data on disease course such as structural damage and extraintestinal manifestations. Fifth, it was beyond the scope of this study to examine medication use. While PPIs and steroids are likely to decrease EoE disease activity, their use may also reflect underlying disease activity. Hence to disentangle the role of treatment and disease activity for the CVD risk of EoE, other study designs such as propensity score models would be needed. Finally, the study population in our sample was mainly of Caucasian origin, limiting the generalizability to other populations.

In conclusion, we found no increased risk of incident MACEs in a cohort of more than 1500 patients with biopsy-proven EoE compared to reference individuals. The results remained unchanged after adjustment for concomitant disease, relevant CVD medications, and when compared with full siblings. The absence of any association of EoE with MACEs in our study is reassuring for patients with EoE.

### Supplementary Information

Below is the link to the electronic supplementary material.

Supplementary file1 (DOCX 65 KB)

## Data Availability

The data underlying this article cannot be shared publicly due to Swedish regulations.

## Abbreviations

aHR: Adjusted hazard ratio
CVD: Cardiovascular disease
COPD: Chronic obstructive pulmonary disease
CI: Confidence interval
CHF: Congestive heart failure
EoE: Eosinophilic esophagitis
ESPRESSO: Epidemiology strengthened by histopathology reports in Sweden
GI: Gastrointestinal
ICD: International classification of diseases
IHD: Ischemic heart disease
MACE: Major adverse cardiovascular events
NPR: National patient register
PPV: Positive predictive value
PY: Person-years
RD: Rate difference
SNOMED: Systematised nomenclature of medicine
