# Supplementary material for: Eosinophilic esophagitis and risk of incident major adverse cardiovascular events: a nationwide matched cohort study
Source: Esophagus. 2024 May 29;21(3):365–73. doi: 10.1007/s10388-024-01066-8 (PMC11199241; doi:10.1007/s10388-024-01066-8)
Supplement: Supplementary file 1 — Supplementary file1 (DOCX 65 KB) [file 10388_2024_1066_MOESM1_ESM.docx]

**Supplementary Material**

This supplement contains additional tables and figures to the study by Anders Forss et al:

"*Eosinophilic esophagitis and risk of incident major adverse cardiovascular events: A nationwide matched histology cohort study*"

**Table of contents**

**Table S1.** Study exclusion criteria (page 2)

**Table S2.** Definitions of primary and secondary outcomes (page 3)

**Table S3.** Definitions of covariates and prescription medications (page 4-5)

**Table S4.** Cohort characteristics in patients with eosinophilic esophagitis and general population reference individuals at the start of follow-up in a restricted cohort 2006-2017 with follow-up until 2019 (pages 6-7)

**Table S5.** Stratified analyses of hazard ratios and incidence rates for incident major adverse cardiovascular events in patients with eosinophilic esophagitis compared to general population reference individuals 1990-2019 (page 8)

**Table S6.** Incidence rates and hazard ratios for incident major adverse cardiovascular events in patients with eosinophilic esophagitis compared to full siblings 1990-2019 (page 9)

**Table S7**. Incidence rates and hazard ratios for incident major adverse cardiovascular events in patients with eosinophilic esophagitis compared to general population reference individuals in a restricted cohort 2006-2019 (page 10)

**Table S8** Stratified analyses with hazard ratios for incident major adverse cardiovascular events in patients with eosinophilic esophagitis compared to general population reference individuals exposed to corticosteroids and proton pump inhibitors in a restricted cohort 2006-2019 (page 11)

**Figure S1**. Flowchart of study exclusion and inclusion 1990-2017 (page 12)

| **Table S1 Study exclusion criteria** | | | | | |
| --- | --- | --- | --- | --- | --- |
|  | **Excluded conditions** |  | **Definition^*^** |  |  |
|  | **Cardiovascular disease** | ICD-7/8 | ICD-9 | ICD-10 |  |
|  |  | 394-399; 410-445; Procedure code op-6; 3065, 3066, 3105, 3127, 3158, 3092, 3080, 3068, 3085, 3149 | 391-400; 406-459; Procedure code op-6; 3065, 3066, 3105, 3127, 3158, 3092, 3080, 3068, 3085, 3149 | I00-I99; G45; Procedure code op-6, op-7;  F procedure codes |  |
|  | | | | | |

**ICD, international classification of disease**

^*^ **Hospital inpatient discharge letters or outpatient specialty care notes in the Swedish National Patient Register. If recorded in the registers on or before the index biopsy date for Eosinophilic esophagitis**

**Table S2** Definitions of primary and secondary outcomes

|  | **Outcome^*^** | **Definition** | **Data source and criteria^#^** |
| --- | --- | --- | --- |
|  | **Primary** |  |  |
|  | Major adverse cardiovascular event | Composite outcome including any of the following secondary outcomes (defined below):  (1) ischemic heart disease (including acute myocardial infarction)  (2) congestive heart failure  (3) stroke  (4) cardiovascular death |  |
|  | **Secondary** |  | Hospital inpatient discharge letters or outpatient specialty care notes in the Swedish National Patient Register. |
|  | Ischemic heart disease |  | ICD-8/9: 410-414; ICD-10: I20-25 |
|  | Congestive heart failure |  | ICD-8: 427,00, 427,10; ICD-9: 428A, 428B, 428X; ICD-10: I11.0, I50 |
|  | Stroke |  | ICD-8/9: 430-438; ICD-10: I60-64 |
|  | Cardiovascular mortality | Cause of Death Register (primary cause of death) | ICD-8: 390-458; ICD-9: 390-459; ICD-10: I00-I99 |

**ICD, international classification of disease**

**^*^ If recorded in the registers after index biopsy date for eosinophilic esophagitis.**

| **Table S3** Definitions of covariates and prescription medications | | | | | |
| --- | --- | --- | --- | --- | --- |
|  |  |  | **Definition** |  |  |
|  | **Comorbidities^*^** | ICD-7/8 | ICD-9 | ICD-10 |  |
|  | Diabetes | 250 | 250 | E10.0-E14.9 |  |
|  | Obesity | 278, 649,1, 649B | 278, 649,1, 649B | E65-66 |  |
|  | Hypertension | - | 400-404; 401-405 | I10-I16 |  |
|  | Dyslipidemia | 272 | 272 | E78 |  |
|  | Chronic kidney disease | 585, 586, Y29,01 | 585, 586, 753B, V42A, V45B, V56 | N18-23; N26; T82.4; Y84.1; Q61; Z49; Z99.2; Z94.0 |  |
|  | Chronic respiratory disease | 490-496 | 490-496 | J40-47 |  |
|  |  |  | Topographic codes | SNOMED codes | Biopsy-proven |
|  | Celiac disease |  | All T64, only T65, T65000, T651 | D6218, D62180, D62188, D6218X, D6218Y; M58, M5800, M58000, M58001, M58005, M58006, M58007 |  |
|  | Atopic dermatitis | 691,00 | 691 | L20.0–L20.9 |  |
|  | Inflammatory bowel disease | 556, 555 | 556, 555 | K50; K51; K52.3 | If ≥2 ICD codes |
|  | **Medication**^†^ |  | **ATC codes** |  |  |
|  | Antiarrhythmics |  | C01B | C01B | Including digitalis. |
|  | Vasodilators |  | C02A-N, C03AA-AB, C03BA, C03CA, C03DA, C03EA, C08DA51, C08DA, C08CA, C08DA, C08DB, C09AB C09BA-BB, C09CA-CB, C09DA, C09DB01 | C01DA,  C07AA, CO7AB, C07AB02, C07BB, C07CB, C07AG01 | Includes both antihypertensives and vasodilators. |
|  | Statins |  | C10 |  |  |
|  | Antithrombotics |  | B01AA, B01AC (except aspirin: B01AC06), B01AE, B01AF, B01AX |  |  |
|  | Proton pump inhibitors |  | A02BC |  |  |
|  | Corticosteroids |  | Systemic:  Swallowed/topical: | H02AB  R03BA01, R03BA02, R03BA05, R03BA08, R01AD09 |  |
|  | | | | | |

ATC, anatomical therapeutic chemical code; ICD, international classification of disease; SNOMED, systematized nomenclature of medicine

**^*^ Hospital inpatient discharge letters or outpatient specialty care notes in the National Swedish Patient Register. If recorded in the registers on or before the index biopsy date for eosinophilic esophagitis.**

^†^ **Recorded in the Prescribed Drug Register 1 January 2006 onwards, and if dispensed on or before index biopsy date for eosinophilic esophagitis.**

| \| **Table S4 Cohort characteristics for patients with eosinophilic esophagitis and general population reference individuals at start of follow-up in a restricted cohort 2006-2017 with follow-up until 2019** \| \| \| \| --- \| --- \| --- \| \| **Characteristics** \| **Reference individuals** \| **EoE** \| \|  \| n (%) \| n (%) \| \| **Total** \| 7 106 \| 1 508 \| \| **Sex** \|  \|  \| \| Male \| 5 336 (75.1) \| 1 137 (75.4) \| \| Female \| 1 770 (24.9) \| 371 (24.6) \| \| **Age** (years) \|  \|  \| \| Mean (SD) \| 35.3 (18.9) \| 36.6 (19.5) \| \| Median (IQR) \| 36 (18-50) \| 37 (19-51) \| \| Range (Min-max) \| 0-90 \| 0-90 \| \| **Age-group** (years) \|  \|  \| \| <18 \| 1 705 (24.0) \| 346 (22.9) \| \| 18 <40 \| 2 265 (31.9) \| 459 (30.4) \| \| 40 <60 \| 2 347 (33.0) \| 498 (33.0) \| \| ≥60 \| 789 (11.1) \| 205 (14.0) \| \| **Country of birth** \|  \|  \| \| Nordic \| 5 930 (83.5) \| 1 433 (95.0) \| \| Other \| 1 175 (16.5) \| 75 (5.0) \| \| NA \| 1 (0.0) \| 0 (0.0) \| \| **Educational level** (years) \|  \|  \| \| Compulsory school (≤9) \| 1 041 (14.7) \| 225 (14.9) \| \| Upper secondary school (10-12) \| 2 506 (35.3) \| 540 (35.8) \| \| College or university (≥13) \| 2 237 (31.5) \| 476 (31.6) \| \| NA \| 1 322 (18.6) \| 267 (17.7) \| \| **Start of follow-up period** \|  \|  \| \| 2006 - 2009 \| 775 (10.9) \| 163 (10.8) \| \| 2010 - 2017 \| 6 331 (89.1) \| 1 345 (89.2) \| \| **Follow-up** (years) \|  \|  \| \| Mean (SD) \| 6.3 (2.5) \| 6.4 (2.5) \| \| Median (IQR) \| 5.9 (4.5-7.8) \| 6.0 (4.6-7.8) \| \| Range, min-max \| 0.0-14.0 \| 0.1-14.0 \| \| 0-1 \| 152 (2.1) \| 25 (1.7) \| \| 2-9 \| 6 238 (87.8) \| 1 335 (88.5) \| \| ≥10 \| 716 (10.1) \| 148 (9.8) \| \| **Comorbidity at start of follow-up** \|  \|  \| \| Any metabolic disease ≥1^*^ \| 383 (5.4) \| 109 (7.2) \| \| Diabetes \| 115 (1.6) \| 30 (2.0) \| \| Obesity \| 107 (1.5) \| 19 (1.3) \| \| Hypertension \| 204 (2.9) \| 67 (4.4) \| \| Dyslipidemia \| 62 (0.9) \| 24 (1.6) \| \| Kidney disease \| 100 (1.4) \| 28 (1.9) \| \| Celiac disease \| 2 (0.03) \| 46 (3.1) \| \| Chronic respiratory disease^†^ \| 384 (5.4) \| 269 (17.8) \| \| Atopic dermatitis \| 146 (2.1) \| 104 (6.9) \| \| Inflammatory bowel disease \| 3 (0.0) \| 39 (3.0) \| \| **Medication during follow-up^‡^** \|  \|  \| \| Antiarrhythmics \| 51 (0.7) \| 27 (1.8) \| \| Antithrombotics \| 76 (1.1) \| 17 (1.1) \| \| Statins \| 348 (4.9) \| 92 (6.1) \| \| Vasodilators \| 741 (10.4) \| 202 (13.4) \|   EoE, eosinophilic esophagitis; IQR, interquartile range; NA, data not available; SD, standard deviation  ^*^ Includes ≥1 of diabetes, obesity, hypertension and dyslipidemia.  ^†^ Includes chronic obstructive pulmonary disease and asthma.  **^‡^** Includes ATC codes in Table S3 |  |  |  |
| --- | --- | --- | --- | --- | --- | --- | --- | --- | --- | --- | --- | --- | --- | --- | --- | --- | --- | --- | --- | --- | --- | --- | --- | --- | --- | --- | --- | --- | --- | --- | --- | --- | --- | --- | --- | --- | --- | --- | --- | --- | --- | --- | --- | --- | --- | --- | --- | --- | --- | --- | --- | --- | --- | --- | --- | --- | --- | --- | --- | --- | --- | --- | --- | --- | --- | --- | --- | --- | --- | --- | --- | --- | --- | --- | --- | --- | --- | --- | --- | --- | --- | --- | --- | --- | --- | --- | --- | --- | --- | --- | --- | --- | --- | --- | --- | --- | --- | --- | --- | --- | --- | --- | --- | --- | --- | --- | --- | --- | --- | --- | --- | --- | --- | --- | --- | --- | --- | --- | --- | --- | --- | --- | --- | --- | --- | --- | --- | --- | --- | --- | --- | --- | --- | --- | --- | --- | --- | --- | --- | --- | --- | --- | --- | --- | --- | --- | --- | --- | --- | --- | --- | --- | --- | --- | --- | --- |
|  |  |  |  |

**Table S5 Stratified analyses of hazard ratios and incidence rates for incident major adverse cardiovascular events in patients with eosinophilic esophagitis compared to general population reference individuals 1990-2019**

| **Category** | **Reference individuals** | **EoE** | |
| --- | --- | --- | --- |
|  | MACE/n (%) | MACE/n (%) | Adjusted HR^*^ (95%CI) |
| **Total** (N=) | 7 281 | 1 546 | |
| **Sex** |  |  |  |
| Male | 173/5 446 (3.2) | 55/1 160 (4.7) | 1.32 (0.97-1.81) |
| Female | 52/1 835 (2.8) | 10/386 (2.6) | 0.52 (0.25-1.08) |
| **Age group** (years) |  |  |  |
| <40 | 8/4 082 (0.2) | 2/828 (0.2) | 0.99 (0.21-4.68) |
| 40 <60 | 98/2 380 (4.2) | 30/505 (5.9) | 1.52 (1.00-2.32) |
| ≥60 | 119/819 (14.5) | 33/213 (15.5) | 0.94 (0.63-1.41) |
| **Follow-up** (years) |  |  |  |
| <5 | 143/2 354 (6.1) | 37/491 (7.5) | 0.98 (0.68-1.42) |
| ≥5 | 82/4 927 (1.7) | 28/1 055 (2.7) | 1.35 (0.85-2.14) |
| **Start of follow-up** |  |  |  |
| 1990 - 2000 | 18/69 (26.0) | 7/16 (43.8) | 1.23 (0.50-3.02) |
| 2001 - 2010 | 43/881 (4.9) | 13/185 (7.0) | 1.58 (0.82-3.04) |
| 2011 - 2017 | 164/6 331 (2.6) | 45/1 345 (3.3) | 1.07 (0.77-1.51) |
| **Country of birth** |  |  |  |
| Nordic | 196/6 087 (3.2) | 63/1 470 (4.3) | 1.13 (0.85-1.52) |
| Other | 29/1 193 (2.4) | 2/76 (2.6) | - |
| **Comorbidity** |  |  |  |
| Any metabolic disease ≥1^†^ | 46/389 (11.8) | 11/110 (10.0) | 2.30 (0.68-7.82) |
| No metabolic disease | 179/6 892 (2.6) | 54/1 436 (3.8) | 1.28 (0.92-1.76) |

CI, confidence interval; EoE, eosinophilic esophagitis; HR, hazard ratio; MACE, major adverse cardiovascular events; PY, person-years

^*^ Adjusted for age, sex, calendar year, county of residence, country of birth (Nordic country or other), educational level (compulsory school, upper secondary school or college/university), ≥1 metabolic disease (diabetes, obesity, hypertension or dyslipidemia), chronic kidney disease, celiac disease, atopic dermatitis and chronic respiratory disease (includes chronic obstructive pulmonary disease and asthma).

^†^ Includes ≥1 diabetes, obesity, hypertension or dyslipidemia.

**Table S6 Incidence rates and hazard ratios for incident major adverse cardiovascular events in patients with eosinophilic esophagitis compared to full siblings 1990-2019**

|  |  | **Full siblings** | **EoE** |
| --- | --- | --- | --- |
|  |  | N=1 958 | N=1 178 |
|  | **MACE*** |  |  |
|  | Incident events (%) | 57 (2.9) | 42 (3.6) |
|  | Incidence rate per 1000 PY (95%CI) | 4.3 (3.3-5.6) | 5.4 (3.9-7.3) |
|  | Absolute rate difference per 1000 PY (95%CI) | 0 (ref.) | 1.08 (-0.9-3.1) |
|  | Unadjusted HR (95%CI) | 1 (ref.) | 1.49 (0.98-2.26) |
|  | Adjusted HR^†^ (95%CI) | 1 (ref.) | 1.33 (0.81-2.16) |

CI, confidence interval; EoE, eosinophilic esophagitis; HR, hazard ratio; MACE, major adverse cardiovascular events; PY, person-years

^*^ Includes ischemic heart disease, congestive heart failure, stroke and cardiovascular mortality.

^†^ Adjusted for age, sex, calendar year, county of residence at index date, country of birth (Nordic country or other), educational level (compulsory school, upper secondary school or college/university), ≥1 metabolic disease (diabetes, obesity, hypertension or dyslipidemia), chronic kidney disease, celiac disease, atopic dermatitis and chronic respiratory disease (includes chronic obstructive pulmonary disease and asthma).

**Table S7 Incidence rates and hazard ratios for incident major adverse cardiovascular events in patients with eosinophilic esophagitis compared to general population reference individuals in a restricted cohort 2006-2019**

|  |  | **Reference individuals** | **EoE** |
| --- | --- | --- | --- |
|  |  | N=7 106 | N=1 508 |
|  | **MACE*** |  |  |
|  | Incident events (%) | 198 | 55 |
|  | Incidence rate per 1000 PY (95%CI) | 4.4 (3.8-5.1) | 5.7 (4.3-7.4) |
|  | Absolute rate difference per 1000 PY (95%CI) | 0 (ref.) | 1.3 (-0.3-3.0) |
|  | Unadjusted HR (95%CI) | 1 (ref.) | 1.30 (0.97-1.75) |
|  | Adjusted HR^†^ (95%CI) | 1 (ref.) | 1.09 (0.80-1.47) |
|  | Adjusted for CVD medication  HR**^‡^** (95%CI) | 1 (ref.) | 1.07 (0.79-1.46) |

CI, confidence interval; CVD, cardiovascular disease; EoE, eosinophilic esophagitis; HR, hazard ratio; MACE, major adverse cardiovascular events; PY, person-years

^*^ Includes ischemic heart disease, congestive heart failure, stroke and cardiovascular mortality.

^†^Adjusted for age, sex, calendar year, county of residence at index date, country of birth (Nordic country or other), educational level (compulsory school, upper secondary school or college/university), ≥1 metabolic disease (diabetes, obesity, hypertension or dyslipidemia), chronic kidney disease, celiac disease, atopic dermatitis and chronic respiratory disease (includes chronic obstructive pulmonary disease and asthma).

**^‡^** Adjusted HR and additional adjustment for four groups of cardiovascular medications: antiarrhythmics, antithrombotics, statins and vasodilators. See Table S3 for ATC codes for the respective group.

**Table S8 Stratified analyses with hazard ratios for incident major adverse cardiovascular events in patients with eosinophilic esophagitis compared to general population reference individuals exposed to corticosteroids and proton pump inhibitors in a restricted cohort 2006-2019**

| **Category** | **Reference individuals** | **EoE** | |  |
| --- | --- | --- | --- | --- |
|  | MACE/n (%) | MACE/n (%) | Adjusted HR^*^ (95%CI) | P-heterogeneity |
| **Total** (N=) | 7 106 | 1 508 | |  |
| **Steroids** |  |  |  |  |
| No steroids | 165/5 659 (2.9) | 44/1 206 (3.6) | 1.04 (0.75-1.45) | 0.52 |
| Steroids (any) | 33/1 447 (2.3) | 11/302 (3.6) | 1.34 (0.67-2.68) |  |
| **PPI** |  |  |  |  |
| No PPIs | 143/5 089 (2.8) | 40/1 085 (3.7) | 1.09 (0.76-1.55) | 0.98 |
| PPIs | 55/2 017 (2.7) | 15/423 (3.5) | 1.08 (0.61-1.91) |  |
| **Combination** |  |  |  |  |
| Neither | 120/4 088 (2.9) | 32/876 (3.7) | 1.02 (0.69-1.51) | 0.50 |
| Steroids and PPIs | 10/446 (2.2) | 3/93 (3.2) | 1.64 (0.43-6.22) |  |
|  |  |  |  |  |

CI, confidence interval; EoE, eosinophilic esophagitis; HR, hazard ratio; MACE, major adverse cardiovascular events; PPIs, proton pump inhibitors; PY, person-years

^*^ Adjusted for age, sex, calendar year, county of residence, country of birth (Nordic country or other), educational level (compulsory school, upper secondary school or college/university), ≥1 metabolic disease (diabetes, obesity, hypertension or dyslipidemia), chronic kidney disease, celiac disease, atopic dermatitis and chronic respiratory disease (includes chronic obstructive pulmonary disease and asthma).

**Figure S1** Flowchart of exclusion and inclusion of patients with biopsy-proven eosinophilic esophagitis in the ESPRESSO (Epidemiology Strengthened by Histopathology Reports) histopathology cohort and matched general population reference individuals from the Swedish Total Population Register 1990-2017.

CVD, cardiovascular disease
